# Hyperhomocysteinemia in hypofertile male patients can be alleviated by supplementation with 5MTHF associated with one carbon cycle support

**DOI:** 10.3389/frph.2023.1229997

**Published:** 2023-08-29

**Authors:** Arthur Clement, Edouard Amar, Patrice Clement, Éric Sedbon, Charles Brami, Silvia Alvarez, Yves Menezo

**Affiliations:** ^1^ Laboratoire Clément, Genetics and IVF, Avenue d'Eylau, Paris, France; ^2^ Cabinet Médical Urology, Andrology, Avenue Victor Hugo, Paris, France; ^3^ Cabinet Médical, Gyn Obst, 17 rue Pétrarque, Paris, France; ^4^ Cabinet Médical, Gyn Obst, 16 Avenue Paul Doumer, Paris, France; ^5^ Cabinet Médical, Gyn Obst, 15 Avenue Pointcarré, Paris, France

**Keywords:** male hypofertility, homocysteine, MTHFR SNP, 5 MTHF, one carbon cycle, folates cycle

## Abstract

**Introduction:**

Homocysteine (Hcy) is a cellular poison, side product of the hydrolysis of S-Adenosyl Homocysteine, produced after the universal methylation effector S -Adenosylmethionine liberates a methyl group to recipient targets. It inhibits the methylation processes and its rising is associated with multiple disease states and ultimately is both a cause and a consequence of oxidative stress, affecting male gametogenesis. We have determined hyper homocysteinhemia (HHcy) levels can be reliably reduced in hypofertile patients in order to decrease/avoid associated epigenetic problems and protect the health of future children, in consideration of the fact that treatment with high doses of folic acid is inappropriate.

**Methods:**

Homocysteine levels were screened in male patients consulting for long-standing infertility associated with at least three failed Assisted Reproductive Technology (ART) attempts and/or repeat miscarriages. Seventy-seven patients with Hcy levels > 15 µM were treated for three months with a combination of micronutrients including 5- MethylTetraHydroFolate (5-MTHF), the compound downstream to the MTHFR enzyme, to support the one carbon cycle; re-testing was performed at the end of a 3 months treatment period. Genetic status for Methylenetetrahydrofolate Reductase (MTHFR) Single nucleotide polymorphisms (SNPs) 677CT (c.6777C > T) and 1298AC (c.1298A > C) was determined.

**Results:**

Micronutrients/5-MTHF were highly efficient in decreasing circulating Hcy, from averages 27.4 to 10.7 µM, with a mean observed decrease of 16.7 µM. The MTHFR SNP 677TT (homozygous form) and combined heterozygous 677CT/1298AC status represent 77.9% of the patients with elevated Hcy.

**Discussion:**

Estimation HHcy should not be overlooked in men suffering infertility of long duration. MTHFR SNPs, especially 677TT, are a major cause of high homocysteinhemia (HHcy). In these hypofertile patients, treatment with micronutrients including 5-MTHF reduces Hcy and even allows spontaneous pregnancies post treatment. This type of therapy should be considered in order to ensure these patients' quality of life and avoid future epigenetic problems in their descendants.

## Introduction

**Figure 1:**
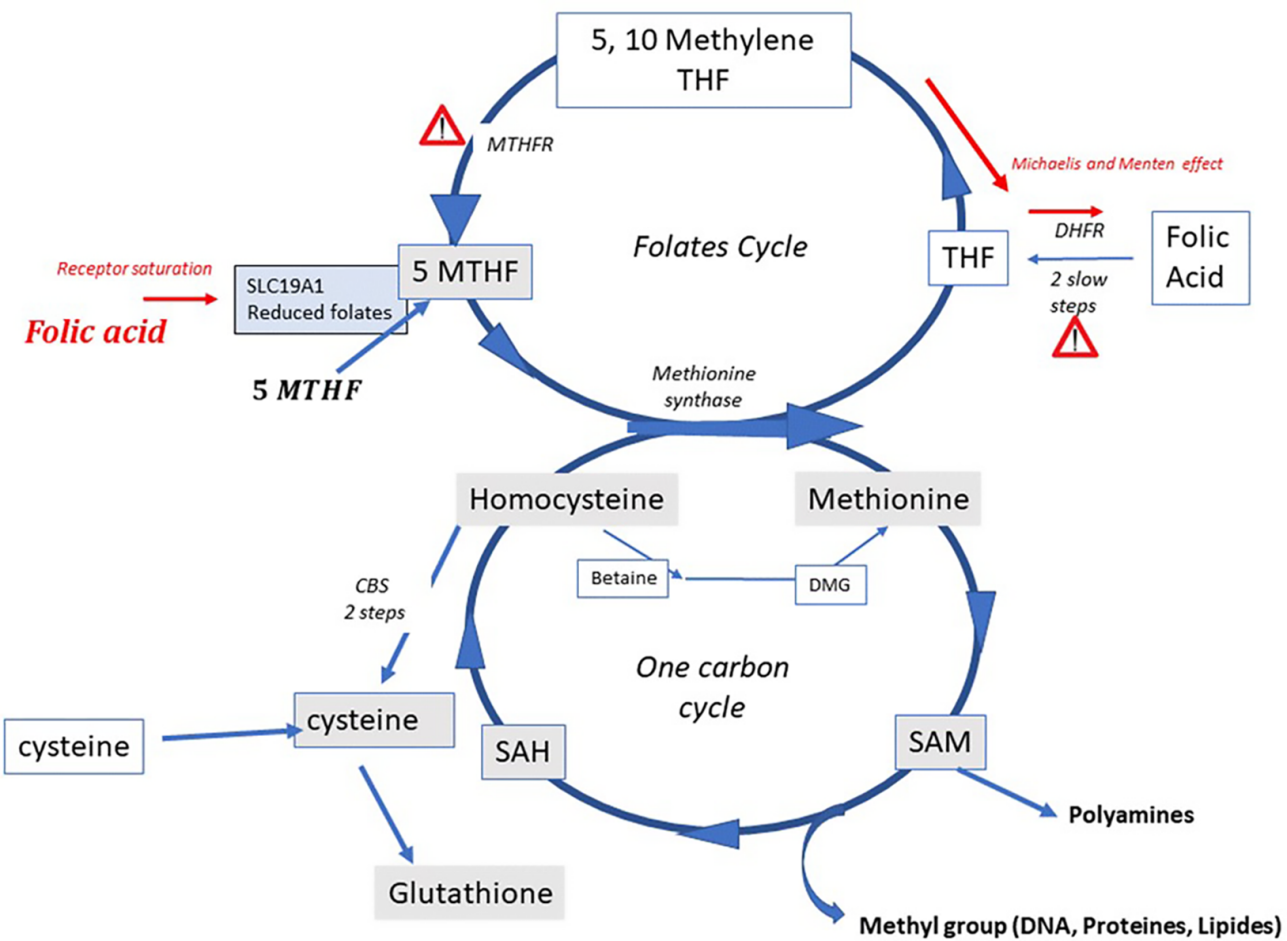
The one carbon and the folate cycles; indicating the MTHFR bottleneck and the two severe Michaelis and Menten effects induced by folic acid (FA) at high doses when excessive unmetabolized metabolite is present upstream from MTHFR: at the level of DHFR (DiHydrofolate reductase) [Bailey and Ayling (4), and at the level of MTHFR itself]. A further negative effect occurs due to saturation of the solute carrier for the natural folate 5MTHF by UMFA (UnMetabolized folic acid).

Homocysteine (Hcy) is a cellular poison that accumulates from hydrolysis of S-Adenosyl Homocysteine, produced after the universal methylation effector S -AdenosylMethionine liberates a methyl group to recipient targets. Elevated Hcy is associated with multiple disease states (1, 2) and ultimately is both a cause and a consequence of oxidative stress (3). It must be recycled to methionine via the One-Carbon Metabolism (OCM, Figure 1) by re-methylation from either of 5-Methyltetrahydrofolate (5-MTHF), resulting from the folates cycle, or from betaine, resulting from choline oxidation in mitochondria. These pathways are subject to numerous polymorphisms that affect their efficiency, the most common being those that affect the MTHFR (Methylene TetraHydroFolate Reductase) enzyme. This enzyme catalyses formation of 5MTHF, the final methyl donor that allows generation of methionine via Methionine Synthase (MS). Homocysteine can also be converted to cysteine via the Cystathionine beta synthase (CBS) pathway. This alleviates the HHcy burden, but jeopardizes the cell's methylation capacity, in particular DNA and histone methylation, resulting in hypomethylation and DNA instability (4–6). Hcy elevation is greater in men than in women, essentially due to androgen inhibition of the CBS pathway and its stimulation by estrogen (7) In terms of fertility, Hcy is related to sperm quality via generation of oxidative stress (8); however, perhaps even more importantly, it inhibits methylation processes (9), known to be regulators of sperm fertility potential/developmental capacity (10–13). As a consequence, there is a risk of transmitting problems associated with epigenetics/imprinting (14–17). Patients affected by MTHFR Single nucleotide polymorphisms (MTHFR SNPs), especially A1298C and C677T, have a reduced capacity to generate 5-MTHF, thus leading to accumulation of Hcy. This has a significant effect on fertility (6, 18–20), either due to decreased enzyme activity directly or in relation to Hcy elevation. Several molecules have been proposed/tested (21) in order to overcome methylation problems and associated epigenetic risks. Treatment with high doses of folic acid is not suitable, as this causes deterioration in the sperm methylation profile (22, 23): folic acid has poor metabolic capacity (4), leading to accumulation of unmetabolized folic acid (UMFA) upstream from MTHFR, with a negative impact on metabolic capacity of the enzyme. It also saturates the receptors for MTHF, the natural product in food, preventing its entry into cells (24, 25). Folinic acid lies upstream from MTHFR and may also accumulate. 5-MTHF, located downstream from the MTHFR enzyme, is the natural molecule that allows methionine to be regenerated from Hcy. In the present study, 77 male patients consulting for infertility of long duration and showing high levels of Hcy [>15 micromolar] were treated for three months with a full dose of 5-MTHF complemented with other micronutrients in support of the pathway and then tested again for Hcy levels. Their MTHFR SNP status was also recorded.

## Materials and methods

### Ethical considerations

In our units, testing for Hcy and MTHFR SNPs has been standard practice for patients with a history of ART (Assisted Reproductive technology) failures and recurrent miscarriage since 2019. The study follows classical ethics guidelines recommended by the French “Agence de BioMedecine” in accordance with the Declaration of Helsinki; no specific approval is required, but all of the tests must be prescribed for patients seeking fertility treatments by certified andrologist, endocrinologist or obstetrician/gynaecologist. The test must be performed in licensed laboratories with additional license for genetic testing. Informed patient consent must be co-signed by the patient, the clinician and the laboratory. Testing for the purpose of building a control group in a fertile population is not permitted. Patients may decline testing. Based upon our first preliminary data as well as scientific literature, we recommended testing for both partners (19, 20, 26).

### Patients and treatments

Homocysteine levels were measured in 77 male patients consulting for long-lasting infertility associated with three failed ART attempts and/or repeat miscarriages referring to the participating clinics during the last 18 months. Patients with HHcy were offered a nutritional support containing a full dose of 5-MTHF (400 µg) and physiologic amounts of the other two physiologic methyl donors, methyl cobalamin and betaine. The supplements also contained other essential co-factors for the pathway, namely vitamin B2, B3 and B6, conjugated zinc and a cysteine donor (Impryl™ Parthenogen, Switzerland; Tetrafolic™, Nurilia, France). Hcy is tested 3 months after the beginning of treatment, then patients can enter ART attempts under nutritional support.

### Genetic testing

The techniques have been previously described (27, 28). Tests are carried out on 5 µl venous blood samples, using the LAMP human MTHFR mutation kit (LaCAR, MDx, Belgium; https://www.lacar-mdx.com/kit/thrombophilic-profile/mthfr-c677t; https://www.lacar-mdx.com/kit/thrombophilic-profile/mthfr-a1298c) based on selective hybridization. Six specific primers covering the locus of the mutation are used for the 677CT SNP. The same protocol was applied for 1298AC SNP, with the same number of specific primers covering the mutation region. Two loop primers are used in both, and the probes used simultaneously amplify the wild type gene. The results were evaluated by comparing the curves obtained by fluorescence: Optic source, 470 and 590 nm dual color led with high quality interference filter (band pass 40 nm); detection optics: photodiodes with HQ interference filters (510–560 nm long pass, 620 nm long pass) The percentage of each SNP status was recorded.

### Homocysteine

The protocol has been previously described^.^ Briefly: fasting blood samples were collected in the morning, and serum Hcy measured using the ViTROS® kit (Ortho Clinical diagnostic Vienna), which allows determination of homocysteine and homocysteine. After a first reduction step homocysteine is then transformed into cystathionine in the presence of cystathionine beta synthase (CBS). Cystathionine is then hydrolyzed and form Hcy, ammonia, and pyruvate. The reduction of Pyruvate to lactate, by lactic acid dehydrogenase is proportional to all the homocysteine is proportional to all the homocysteine present, and is quantified by the amount of NAD + produced (measured by spectrophotometry at 340 nm) The assay is linear from 1 to 90 µM-homocysteine. There is no real consensus for minimal Hcy cut-off values (10 to 15 µM). We chose a level of 15 μmoles/L as the cut-off for increased risk.

### Statistics

The paired *t*-test was used to determine the difference between the same variable (Hcy) in the same sample of patients before and after treatments (Chi consulting® program).

## Results

**Figure 2:**
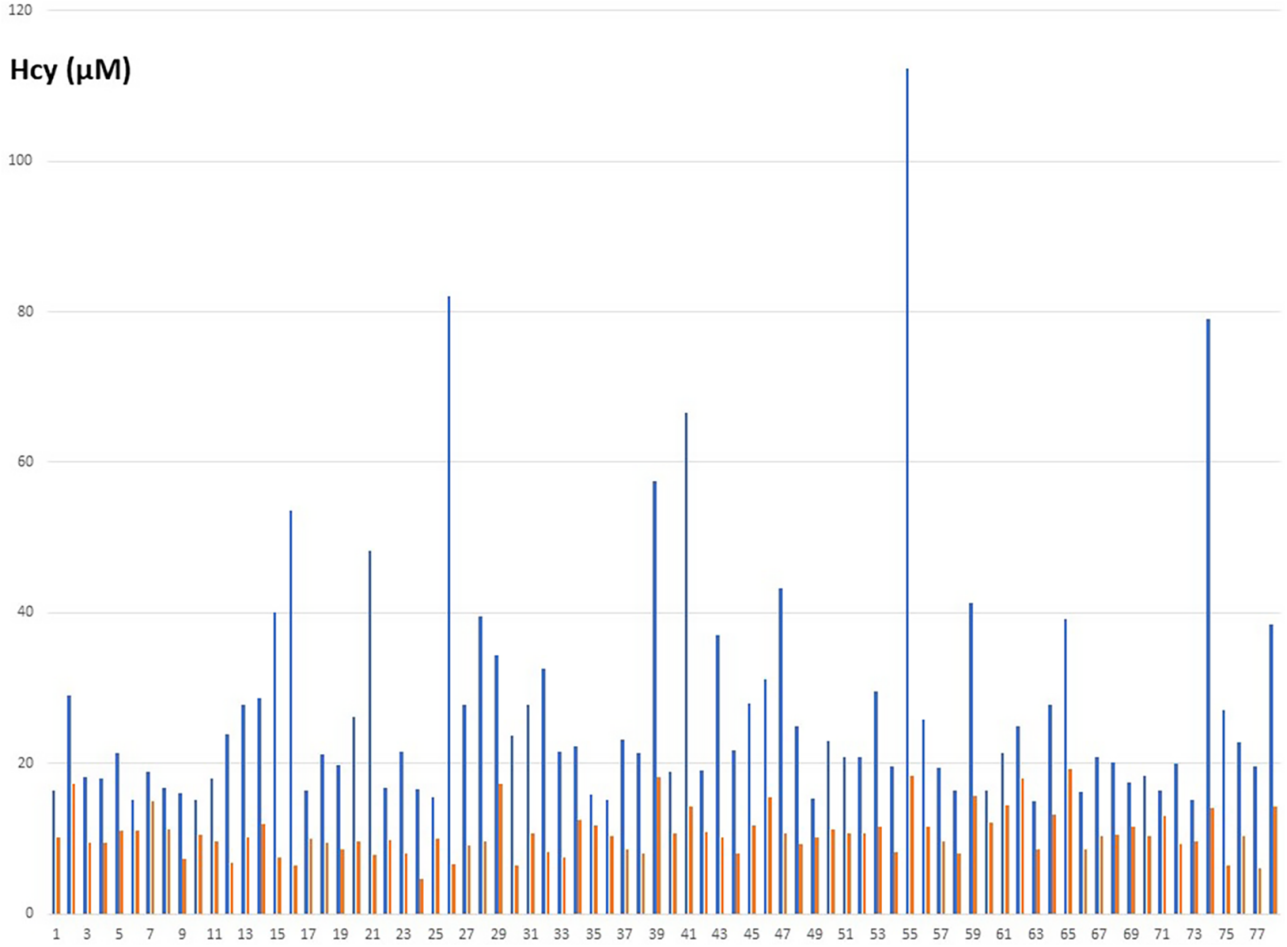
Individual variations of the Hcy concentrations in blood (µM), before (blue) and after (orange) treatment.

**Figure 3:**
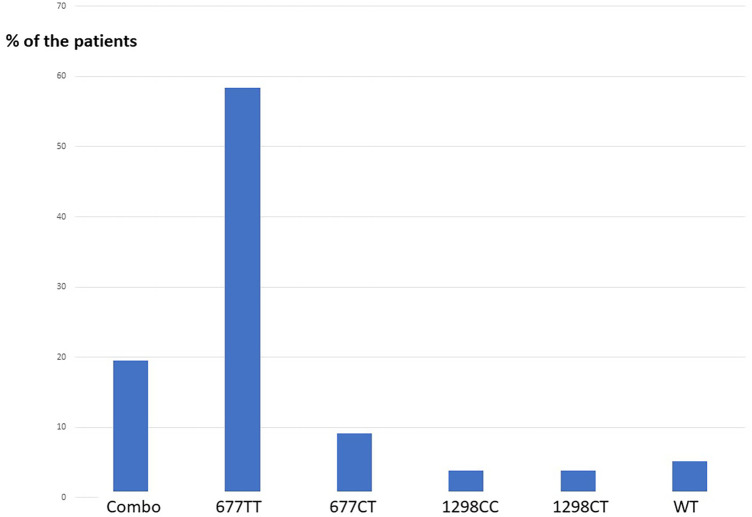
Distribution of 677CT and 1298AC combinations in our population, men with HHcy >15 µM). Combo: double Heterozygoty 677CT/1298AC; WT: wild type patients 677CC/1298AA, no mutation.

Seventy-seven patients were diagnosed high Hcy and were prescribed the treatment. Circulating Hcy levels decreased post-treatment from an average 27.4 µM [SD: 16.7; range 15.2–112,3] to 10.7 µM [SD: 3.1; range 4.2–18.3] with a mean drop of 16 µM (t = 9.2 p: over 10^−10^) (Table 1). The homocysteine level decreased in all the patients assuming the supplement (Figure 2). The distribution of MTHFR SNPs is illustrated in Figure 3: 677TT: 58.4%, 677CT: 9.1%, Combined Heterozygoty (combo) 677CT/1298AC: 19.5%; 1298AC and 1298CC both at 3.9% and 5.2% for the wild type patients. The prevalence of the 677 T mutated allele (patients having at least one 677 T mutated allele) reaches 87%. HHcy decreased from 32.2 to 10.3 µM for the homozygous 677TT patients and from 20.4 to 10.4 µM in the combo 1298AC/677CT patients.

**Table 1:** Variations in circulating homocysteine, post treatment with 5 MethylFolate associated with a support of the one carbon cycle in 77 patients.

Homocysteine (µM)	Mean	SD
Before treatment	27.4	16.7
Post treatment	10.7	3.1
Overall decrease observed	16.7 (−61%)	

### Patient follow up

Results are available for 67 patients; some are waiting for or entering IVF/ICSI programs. Our preliminary results include 15 pregnancies/deliveries, 22%; eight of the pregnancies occurred spontaneously and two resulted in miscarriage.

## Discussion and conclusions

Five major conclusions can be drawn.

1. HHcy in hypofertile males is not “anecdotic” (8, 27). Hcy is well-known to induce pathology, including cancers (29), cardiovascular disease (30), dementia (31), and with an impact on health in general (1, 2). Its role in infertility with respect to the female gamete is well documented (32–34). In men, a direct correlation between Hcy and hypofertility is starting to be recognized (8). Homocysteine and SAH are both strong inhibitors of methylation. Differently from women for whom major methylation occurs during the final stages of follicular growth and oocyte maturation, the final steps of sperm maturation need continuous methylation support (14, 15, 35–37). The negative impact on methylation is also explained by the competition of met and Hcy on the same transporter leading to a decreased methylation index (38)
2. The secondary impact of Hcy involves its causal effect on oxidative stress “Homocysteine*: cause and consequence of Oxidative stress*” (3), this is especially true in men, as male hormones inhibit the CBS pathway (see Figure 1) that generates cysteine from Hcy, subsequently allowing glutathione synthesis. Amongst other associations, this provides a link between methylation defects and oxidative stress in sperm (10, 11). Hcy and cysteine competes also on the same transporters (38).
3. The HHcy in our patients is largely dependent upon the MTHFR SNP 677CT, carried by more than 85% of them, which is in agreement with scientific literature related to cardiovascular and psychiatric disorders. This vouches for 5MTHF as the key player in the positive effect from our treatment. However, we decreased homocysteine also in ten patients not carrying the 677CT variant (4 of them wild type), from 19.4 µM to 12.1 µM for (significant, *p* < 0.0001). These patients are likely carriers of other genetic weaknesses not tested in the present study and affecting other genes of the pathway, whose SNPs may as well occur in double digit prevalence (37) Our results, i.e. 100% response rate, are backed by those from Schiuma et al. (39) they tested the same treatment in women with PCOS and reported decrease of circulating homocysteine in all the exposed patients. On this basis, although 5MTHF seems to play a major role, the availability of a full support to all the 3 pathways for homocysteine removal provided by the treatment may give advantages over plain folate administration by enlarging the rate of responding patients. Moreover, the association of vitamin B12, in its soluble and active methylated form, is of particular relevance, say mandatory, to avoid the so-called folate trap: Briefly, without B12, 5MTHF is not able to release the methyl group to homocysteine, accumulates and does not generate THF, essential for the synthesis of nucleic acids (necessary for DNA repair activity), leading to macrocytic anemia.
4. Homocysteine also has toxic effects on mitochondria, at least in part due to its competition with methionine (40–42), but also to its generation of oxidative stress (3) Mitochondrial is the main effector of energy metabolism drastically involved in sperm motility and vitality; Low Hcy regulation during spermatogenesis is mandatory. However paternal mitochondria are rapidly degraded in the oocyte/early embryo, by autophagy/allophagy, post fertilization.
5. 5-MTHF is a potent and efficient means of decreasing Hcy; this is reinforced by the fact that spontaneous pregnancies/deliveries (>10%) can be achieved post treatment. However, safety is another concern with these treatments, especially if long lasting treatments are in place. Noteworthy, we achieved homocysteine reduction in all our patients by using physiological doses of 5-MTHF, which is a clear-cut advantage compared to the administration of high doses of Folic acid (FA). Indeed, FA has a weak capacity to be metabolized to the physiologic, soluble and active methylated form because requiring a double reduction by DHFR (see Figure 1) and results in the occurrence in circulation of unmetabolized folic acid (UMFA: UMFA syndrome) (4). Excess of FA/UMFA creates negative feedback in the activity of the folate cycle, due to a Michaelis and Menten effect, linked to substrate overloading. It may also lead to saturation of the folate receptor SLC 19A1, which then impairs entry of the “natural” folate MTHF from food intake. As a matter of fact, high doses of FA may mimic a syndrome of total folate deprivation (21, 25, 43, 44), potentially jeopardizing the quality/stability of the germ line (24, 45): the negative impact of FA excess on sperm methylation has been described, which may impact the epigenetic programming of gametes, especially in carriers of MTHFR SNPs (22, 23). 5-Methyltetrahydrofolate does not induce UMFA syndrome (46). However, the “normality” of sperm methylation profile is still to be determined and may vary with epigenetic modifications linked to environment.
  The Rotterdam Periconception Cohort (26) recently recommended that both partners in a couple seeking pregnancy should be tested for homocysteine levels. Our work is in complete agreement with this strategy. In those with increased homocysteine, 5 MTHF should be recommended in order to achieve full efficacy and to avoid metabolic and epigenetic perturbations, thus protecting the health of future generations. The association to 5MTHF of (methyl)B12 and of other micronutrients in support to the pathway is likely to increase the efficacy, to enlarge the rate of responding patients and to avoid safety issues including the folate trap syndrome.

## Data Availability

The original contributions presented in the study are included in the article/Supplementary Material, further inquiries can be directed to the corresponding author.

## References

[1] Koklesova L Mazurakova A Samec M Biringer K Samuel SM Büsselberg D Homocysteine metabolism as the target for predictive medical approach, disease prevention, prognosis, and treatments tailored to the person. EPMA J. (2021) 12:477–505. 10.1007/s13167-021-00263-0 34786033 PMC8581606

[2] Škovierová H Vidomanová E Mahmood S Sopková J Drgová A Cervenová T The molecular and cellular effect of homocysteine metabolism imbalance on human health. Int J Mol Sci. (2016) 17:E1733. 10.3390/ijms17101733 PMC5085763 27775595

[3] Hoffman M. Hypothesis: hyperhomocysteinemia is an indicator of oxidant stress. Med Hypotheses. (2011) 77:1088–93. 10.1016/j.mehy.2011.09.009 21963358

[4] Bailey SW Ayling JE. The extremely slow and variable activity of dihydrofolate reductase in human liver and its implications for high folic acid intake. Proc Natl Acad Sci U S A. (2009) 106:15424–9. 10.1073/pnas.0902072106 19706381 PMC2730961

[5] Duthie S Narayanan S Brand G Pirie L Grant G. Impact of folate deficiency on DNA stability. J Nutr. (2002) 132(Suppl. 8):2444S–9S. 10.1093/jn/132.8.2444S 12163709

[6] Enciso M Sarasa J Xanthopoulou L Bristow S Bowles M Fragouli E Polymorphisms in the MTHFR gene influence embryo viability and the incidence of aneuploidy. Hum Genet. (2016) 135:555–68. 10.1007/s00439-016-1652-z 27068821

[7] Giltay EJ Hoogeveen EK Elbers JM Gooren LJ Asscheman H Stehouwer CD. Effects of sex steroids on plasma total homocysteine levels: a study in transsexual males and females. J Clin Endocrinol Metab. (1998) 83:550–3. 10.1210/jcem.83.2.4574 9467573

[8] Aitken RJ Flanagan HM Connaughton H Whiting S Hedges A Baker MA. Involvement of homocysteine, homocysteine thiolactone, and paraoxonase type 1 (PON-1) in the etiology of defective human sperm function. Andrology. (2016) 4:345–60. 10.1111/andr.12157 26825875

[9] James SJ Melnyk S Pogribna M Pogribny IP Caudill MA. Elevation in S-adenosylhomocysteine and DNA hypomethylation: potential epigenetic mechanism for homocysteine-related pathology. J Nutr. (2002) 132(8 Suppl):2361S–2366. 10.1093/jn/132.8.2361S 12163693

[10] Tunc O Tremellen K. Oxidative DNA damage impairs global sperm DNA methylation in infertile men. J Assist Reprod Genet. (2009) 26:537–44. 10.1007/s10815-009-9346-2 19876730 PMC2788683

[11] Menezo YJ Silvestris E Dale B Elder K. Oxidative stress and alterations in DNA methylation: two sides of the same coin in reproduction. Reprod Biomed Online. (2016) 33:668–83. 10.1016/j.rbmo.2016.09.006 27742259

[12] Marques CT Carvalho F Sousa M Barros A. Genomic imprinting in disruptive spermatogenesis. Lancet. (2004) 363:1700–2. 10.1016/S0140-6736(04)16256-9 15158633

[13] Kobayashi H Sato A Otsu E Hiura H Tomatsu C Utsunomiya T Aberrant DNA methylation of imprinted loci in sperm from oligospermic patients. Hum Mol Genet. (2007) 16:2542–51. 10.1093/hmg/ddm187 17636251

[14] Jenkins TG Aston KJ Pflueger C Cairns BR Carrell DT. Age associated sperm DNA methylation alterations: possible implications in offspring disease susceptibility. PLoS Genet. (2014) 10:e1004458. 10.1371/journal.pgen.1004458 25010591 PMC4091790

[15] Jenkins TG Aston KJ Meyer TD Hotaling JM Shamsi MB Johnstone EB. Decreased fecundity and sperm DNA methylation patterns. Fertil Steril. (2016) 105:51. 10.1016/j.fertnstert.2015.09.013 26453269 PMC4890464

[16] Illum LHR Bak ST Lund S Nielsen AL. DNA Methylation in epigenetic inheritance of metabolic diseases through the male germ line. J Mol Endocrinol. (2018) 60:R39–56. 10.1530/JME-17-0189 29203518

[17] Rotondo JC Lanzillotti C Mazziotta C Tognon M Martini F. Epigenetics of male infertility: the role of DNA methylation. Front Cell Dev Biol. (2021) 9:689624. 10.3389/fcell.2021.689624 34368137 PMC8339558

[18] Gong M Dong W He T Shi Z Huang G Ren R MTHFR 677C> T polymorphism increases the male infertility risk: a meta-analysis involving 26 studies. PLoS One. (2015) 10:e0121147. 10.1371/journal.pone.0121147 25793386 PMC4368707

[19] Jacquesson-Fournols L Alvarez S Cohen M Clement P Menezo Y. A paternal effect of MTHFR SNPs on gametes and embryos should not be overlooked: case reports. J Assist Reprod Genet. (2019) 36:1351–3. 10.1007/s10815-019-01488-9 31119439 PMC6642231

[20] Yu Y Jia C Shi Q Zhu Y Liu Y. Hyperhomocysteinemia in men with a reproductive history of fetal neural tube defects: three case reports and literature review. Medicine. (2019) 98:e13998. 10.1097/MD.0000000000013998 30633186 PMC6336619

[21] Menezo Y Elder K Clement A Clement P. Folic acid, folinic acid, 5 methyl TetraHydroFolate supplementation for mutations that affect epigenesis through the folate and one-carbon cycles. Biomolecules. (2022) 12:197. 10.3390/biom12020197 35204698 PMC8961567

[22] Aarabi M San Gabriel MC Chan D Behan NA Caron M Pastinen T High-dose folic acid supplementation alters the human sperm methylome and is influenced by the MTHFR C677T polymorphism. J Hum Mol Genet. (2015) 24:6301–13. 10.1093/hmg/ddv338 PMC4614702 26307085

[23] Aarabi M Christensen KE Chan D Leclerc D Landry M Ly L Testicular MTHFR deficiency may ex-plain sperm DNA hypomethylation associated with high dose folic acid supplementation. J Hum Mol Genet. (2018) 27:1123–35. 10.1093/hmg/ddy021 PMC6159534 29360980

[24] Menezo Y Clement P Elder K. Are UMFA (un-metabolized folic acid) and endocrine disruptor chemicals (EDCs) co-responsible for sperm degradation? An epigenetic/methylation perspective. Andrologia. (2022) 54:e14400. 10.1111/and.14400 35274767 PMC9541233

[25] Alnabbat KI Fardous AM Cabelof DC Heydari AR. Excessive folic acid mimics folate deficiency in human lymphocytes. Curr Issues Mol Biol. (2022) 44:1452–62. 10.3390/cimb44040097 35723355 PMC9164024

[26] Rubini E Snoek KM Schoenmakers S Willemsen SP Sinclair KD Rousian M First trimester maternal homocysteine and embryonic and fetal growth: the rotterdam periconception cohort. Nutrients. (2022) 14:1129. 10.3390/nu14061129 35334786 PMC8953595

[27] Clément A Amar E Brami C Clément P Alvarez S Jacquesson-Fournols L MTHFR SNPs (methyl tetrahydrofolate reductase, single nucleotide polymorphisms) C677T and A1298C prevalence and serum homocysteine levels in >2100 hypofertile caucasian male patients. Biomolecules. (2022) 12:1086. 10.3390/biom12081086 36008980 PMC9405832

[28] Clément A Chouteau J Clément P Ménézo Y. Importance of the determination of MTHFR SNPs (methylene tetrahydrofolate reductase single nucleotide polymorphisms) in couple infertility. Gynecol Obstet Fertil Senol. (2020) 48:422–7. 10.1016/j.gofs.2020.02.015 32145452

[29] Hasan T Arora R Bansal AK Bhattacharya R Sharma GS Singh LR. Disturbed homocysteine metabolism is associated with cancer. Exp Mol Med. (2019) 51:1–13. 10.1038/s12276-019-0216-4 30804341 PMC6389897

[30] Klerk M Verhoef P Clarke R Blom HJ Kok FJ Schouten EG. MTHFR 677C–>T Polymorphism and risk of coronary heart disease: a meta-analysis. JAMA. (2002) 288:2023–31. 10.1001/jama.288.16.2023 12387655

[31] Smith AD Refsum H. Homocysteine, B vitamins, and cognitive impairment. Annu Rev Nutr. (2016) 36:211–39. 10.1146/annurev-nutr-071715-050947 27431367

[32] Boxmeer JC Macklon NS Lindemans J Beckers NG Eijkemans MJ Laven JS IVF outcomes are associated with biomarkers of the homocysteine pathway in monofollicular fluid. Hum Reprod. (2009) 24:1059–66. 10.1093/humrep/dep009 19221098

[33] Berker B Kaya C Ayta R Satiroglu H. Homocysteine concentrations in follicular fluid are associated with poor oocyte and embryo qualities in polycystic ovary syndrome patients undergoing assisted reproduction. Hum Reprod. (2009) 24:2293–302. 10.1093/humrep/dep069 19443458

[34] Razi Y Eftekhar M Fesahat F Dehghani Firouzabadi R Razi N Sabour M Concentrations of homocysteine in follicular fluid and embryo quality and oocyte maturity in infertile women: a prospective cohort. J Obstet Gynaecol. (2021) 41:588–93. 10.1080/01443615.2020.1785409 32749170

[35] Tian M Bao H Martin FL Zhang J Liu L Huang Q Association of DNA methylation and mitochondrial DNA copy number with human semen quality. Biol Reprod. (2014) 91:101. 10.1095/biolreprod.114.122465 25210131

[36] Poplinski A Tuttelmann F Kanber D Horsthemke B Gromoll J. Idiopathic male infertility is strongly associated with aberrant methylation of MEST and IGF2/H19 ICR1. Int J Androl. (2010) 33:642–9. 10.1111/j.1365-2605.2009.01000.x 19878521

[37] Dattilo M D'Amato G Caroppo E Ménézo Y. Improvement of gamete quality by stimulating and feeding the endogenous antioxidant system: mechanisms, clinical results, insights on gene-environment interactions and the role of diet. J Assist Reprod Genet. (2016) 33:1633–48. 10.1007/s10815-016-0767-4 27423667 PMC5171888

[38] Menezo Y Khatchadourian C Gharib A Hamidi J Greenland T Sarda N. Regulation of S-adenosyl methionine synthesis in the mouse embryo. Life Sci. (1989) 44:1601–9. 10.1016/0024-3205(89)90455-4 2733543

[39] Schiuma N Costantino A Bartolotti T Dattilo M Bini V Aglietti MC Micronutrients in support to the one carbon cycle for the modulation of blood fasting homocysteine in PCOS women. J Endocrinol Invest. (2020) 43:779–86. 10.1007/s40618-019-01163-x 31845191 PMC7230049

[40] Chen LT Xu TT Qiu YQ Liu NY Ke XY Fang L Homocysteine induced a calcium-mediated disruption of mitochondrial function and dynamics in endothelial cells. J Biochem Mol Toxicol. (2021) 35:e22737. 10.1002/jbt.22737 33751715

[41] Zhang T Huang D Hou J Li J Zhang Y Tian M High concentration homocysteine inhibits mitochondrial respiration function and production of reactive oxygen species in neuron cells. Cerebrovasc Dis. (2020) 29:105109. 10.1016/j.jstrokecerebrovasdis.2020.105109 32912537

[42] Lu S Hoestje SM Choo E Epner DE. Induction of caspase-dependent and -independent apoptosis in response to methionine restriction. Int J Oncol. (2003) 22:415–20. 10.3892/ijo.22.2.415 12527942

[43] Cornet D Clement A Clement P Menezo Y. High doses of folic acid induce a pseudo-methylenetetrahydrofolate syndrome. SAGE Open Med Case Rep. (2019) 7:2050313X19850435. 10.1177/2050313X19850435 31205715 PMC6537060

[44] Aguilar-Lacasaña S López-Flores I González-Alzaga B Giménez-Asensio MJ Carmona FD Hernández AF Methylenetetrahydrofolate reductase (MTHFR) gene polymorphism and Infant's anthropometry at birth. Nutrients. (2021) 13:831. 10.3390/nu13030831 33802362 PMC7998581

[45] Cao X Xu J Lin YL Cabrera RM Chen Q Zhang C Excess folic acid intake increases DNA de novo point mutations. Cell Discov. (2023) 9:22. 10.1038/s41421-022-00512-0 36849450 PMC9970956

[46] Prinz-Langenohl R Brämswig S Tobolski O Smulders YM Smith DE Finglas PM 5-MTHF increases plasma folate more effectively than folic acid in women with the homozygous or wild-type6777C ->T polymorphism of MTHFR. Br J Pharmacol. (2009) 158:2014–21. 10.1111/j.1476-5381.2009.00492.x 19917061 PMC2807663

